# En Bloc Resection of Right Renal Cell Carcinoma and Inferior Vena Cava Tumor Thrombus Without Caval Reconstruction: Is It Safe to Divide the Left Renal Vein?

**DOI:** 10.3389/fonc.2022.877310

**Published:** 2022-06-30

**Authors:** Laura Horodyski, Javier Gonzalez, Marina M. Tabbara, Jeffrey J. Gaynor, Maria Rodriguez-Cabero, Felipe Herranz-Amo, Carlos Hernández, Rushi Shah, Gaetano Ciancio

**Affiliations:** ^1^ Department of Urology, University of Miami Miller School of Medicine, Miami, FL, United States; ^2^ Servicio de Urologia, Hospital General Universitario Gregorio Marañón, Madrid, Spain; ^3^ Department of Surgery, University of Miami Miller School of Medicine, Miami, FL, United States; ^4^ Miami Transplant Institute, University of Miami Miller School of Medicine, Jackson Memorial Hospital, Miami, FL, United States

**Keywords:** nephrectomy, inferior vena cava thrombectomy, oncology, tumor thrombus extending to inferior vena cava, renal cell carcinoma

## Abstract

**Introduction:**

It has been suggested that inferior vena cava (IVC) reconstruction following resection of retroperitoneal tumors with IVC tumor thrombus (TT) is not required when adequate collateral circulation is present. There are no reports evaluating mid-term effects on renal function in these patients. The purpose of this study was to assess renal function after *en bloc* resection of right renal cell carcinoma (RCC) with obstructing IVC TT and the possible risks that may arise after left renal vein division.

**Materials and Methods:**

A bi-institutional retrospective review was performed over a 15-year period, assessing patients with right RCC and obstructing level II–IV TT. All patients underwent extensive evaluation and cardiology clearance, and informed consent was obtained for right radical nephrectomy and thrombectomy with or without IVC reconstruction with possible cardiopulmonary bypass (CPB). Patient demographics, tumor characteristics, intraoperative factors, complications, length of stay, and patient survival were evaluated. Preoperative creatinine was recorded, as was creatinine on the day of discharge and at 6 and 12 months postoperatively.

**Results:**

Twenty-two patients were included in the study. Median age at surgery was 62.5 (range: 45–79) years, and 19 (86%) of the patients were men. One patient (5%) had a level II thrombus, 14 patients (64%) had a level III thrombus (IIIa, n = 3; IIIb, n = 6; IIIc, n = 3; IIId, n = 2), and seven patients (32%) had a level IV thrombus. Intraoperatively, median estimated blood loss was 1.35 (range: 0.2–25) L. The median length of hospital stay was 11 (range: 5–50) days. Median preoperative creatinine was 1.20 (range: 0.40–2.70) mg/dl, and postoperatively, median creatinine was 1.3 (range: 0.86–2.20) mg/dl. Median creatinine levels at 6 months and 12 months postoperatively were 1.10 (range: 0.5–1.8) mg/dl and 1.40 (range: 0.6–2.0) mg/dl, respectively. Four patients died (range: 0.1–1.3 years), and median postoperative follow-up among the 18 ongoing survivors (at last follow-up) was 1.5 (range: 0.5–7.0) years.

**Conclusions:**

Resection of right RCC with an obstructing level II–IV TT without reconstruction of the IVC appears to not have a significant adverse effect on mid-term renal function after division of the left renal vein.

## Introduction

Renal cell carcinoma (RCC) infrequently extends into the renal vein and inferior vena cava (IVC) in the form of a tumor thrombus (TT) (1, 2). The classic presentation of flank pain, hematuria, and palpable abdomen is uncommon, with 70% of cases detected incidentally by imaging to investigate nonspecific symptoms (3). Supradiaphragmatic TT formation and the development of distant metastasis are significant adverse prognostic factors (4). Surgical resection is the mainstay treatment of this complex tumor to aid in oncologic control and/or symptomatic palliation (5, 6). As the TT grows, it gradually occludes the IVC lumen, provoking a redistribution of venous blood flow through dilatation of smaller lumbar, azygous, and hemiazygos vessels (7–9).

A few case reports of retroperitoneal masses including sarcomas, Wilms tumor, RCC, and testicular tumors with TT and IVC obstruction necessitating IVC resection have suggested that IVC reconstruction is not required when adequate collateral circulation is present, although a transient rise in creatinine may occur postoperatively (10–12). This may be the result of hemodynamic changes or volume depletion during surgery, in which case improvement is expected over days to weeks after surgery. There are no reports evaluating mid-effects on renal function in these patients. The objective of this study was to assess the impact of *en bloc* resection of right RCC and IVC TT without caval reconstruction on postoperative renal function and the safety and possible risks associated with left renal vein division.

## Materials and Methods

**Figure 1:**
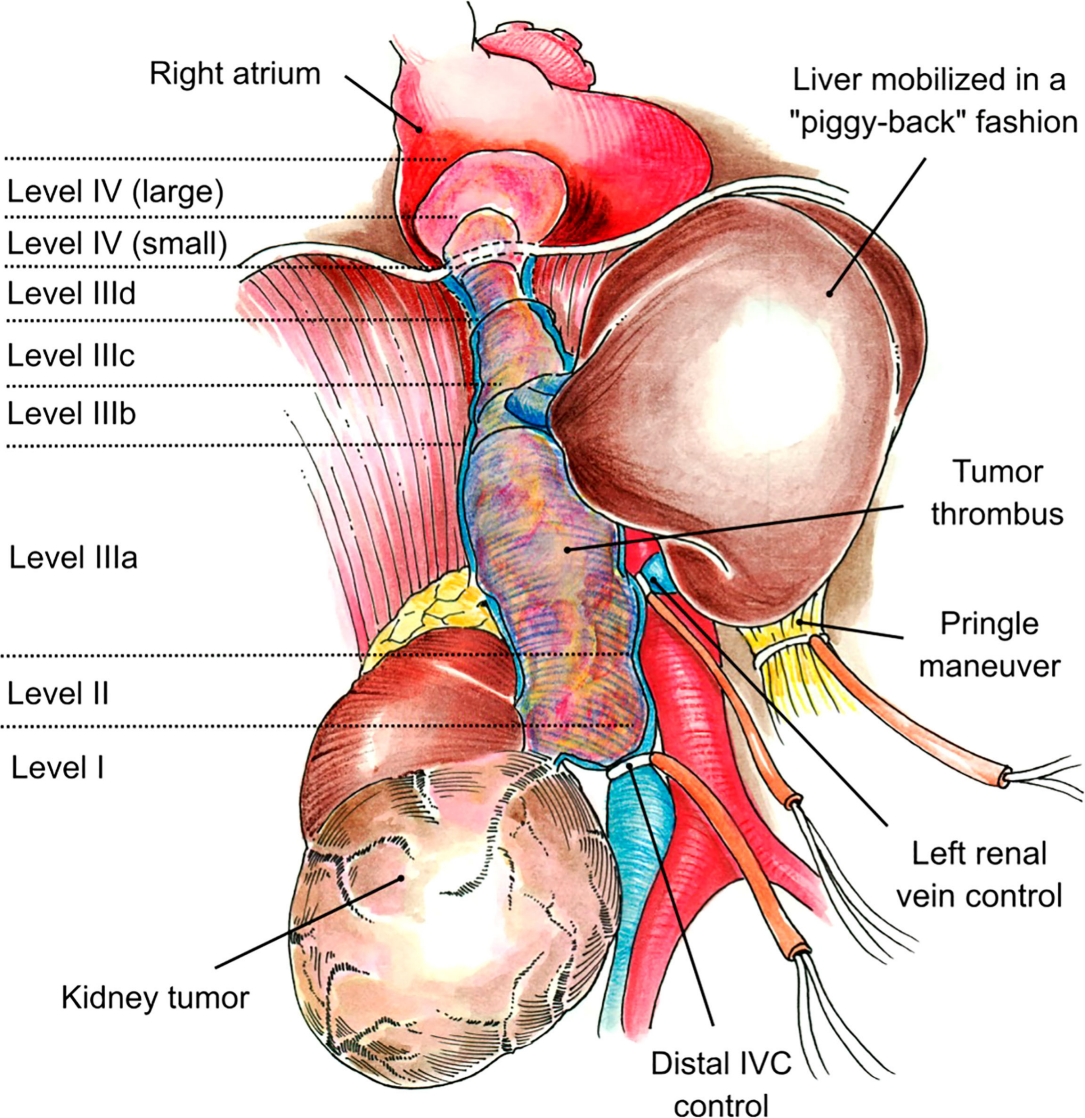
Neves and Zincke Classification System was used for level II and IV thrombus. Level III thrombi were classified as level IIIa (retrohepatic), level IIIb (hepatic), level IIIc (suprahepatic, infradiaphragmatic), or level IIId (supradiaphragmatic, infra-atrial).

A bi-institutional retrospective review was performed for patients undergoing right radical nephrectomy and *en bloc* resection of the IVC and TT between 2005 and 2020. Only patients with obstructing thrombus levels II–IV were included. For level II and IV thrombi, we utilized the classification by Neves and Zincke (13). The cranial extent of the tumor for level III thrombus was defined per our own classification (14). Level III thrombi were classified as level IIIa (retrohepatic), level IIIb (hepatic), level IIIc (suprahepatic, infradiaphragmatic), or level IIId (supradiaphragmatic, infra-atrial) (**Figure 1**) (14).

After extensive evaluation and cardiology clearance, informed consent was obtained for right radical nephrectomy and thrombectomy with or without IVC reconstruction with possible cardiopulmonary bypass (CPB). The cardiothoracic team was on standby for all cases, and CPB was planned in advance for cases of level IV thrombus with a large intra-atrial component.

This study was approved by the University of Miami Miller School of Medicine Institutional Review Board and the Hospital General Universitario Gregorio Marañón Institutional Review Board and follows the ethical principles (as revised in 2013) of the Helsinki Declaration.

### Statistical Analysis

Data collected included patient demographics; tumor characteristics including size, TT level, pathology, grade, and stage; intraoperative factors such as estimated blood loss (EBL), blood transfusions, use of CPB; complications; length of stay; and patient survival. Preoperative creatinine was recorded, as was creatinine on the day of discharge and at 6 and 12 months of postoperative follow-up. When the creatinine level was not available at the exact follow-up time point (6 or 12 months), the value at the time closest to the intended time was used, so long as it was within a 3-month window (thus, for the 6-month value, the 3–9-month measured creatinine level could be included, and similarly, the 9–15-month creatinine level could be included for the 12-month value).

Percentages of patients having selected baseline characteristics were determined as well as means, standard errors, medians, and ranges of values for baseline continuous variables. Deaths that occurred during postoperative follow-up were recorded along with the underlying cause of death. Medians and ranges of values for selected variables were reported as descriptive statistics for patients in this study. In addition, we performed Student’s t-tests of the mean change in the ranks of the differences between the baseline serum creatinine and the serum creatinine at 3 time points: immediate postoperative, 6 months postoperative, and 12 months postoperative serum creatinine levels; these statistical tests are nonparametric and equivalent to the Wilcoxon signed-rank test.

### Surgical Technique

Following the technique previously described by Ciancio et al. (1, 2, 5, 14, 15), a modified Chevron or J-shaped Makuuchi incision was used to gain abdominal access. A liver surgery self-retaining retractor (Rochard or Omni-Tract^®^) was used to create enough space at the level of diaphragmatic domes to further facilitate the approach to the suprahepatic segment of the IVC.

The right renal artery was identified, ligated, and divided by creating a posterior plane of dissection at the level of the inter-aortocaval sulcus (15). After its division, the collateral venous circulation collapsed, making the remaining dissection easier to perform. The liver was completely mobilized off the IVC, with the only remaining structural attachments being the major hepatic veins (piggyback liver mobilization) and the liver hilum (1, 2, 5, 15). A plane was then created between the IVC and the posterior abdominal wall to obtain circumferential control of the IVC. At this level, the engorged small tributaries were properly identified and ligated to prevent significant bleeding and to facilitate stapling of the IVC.

**Figure 2:**
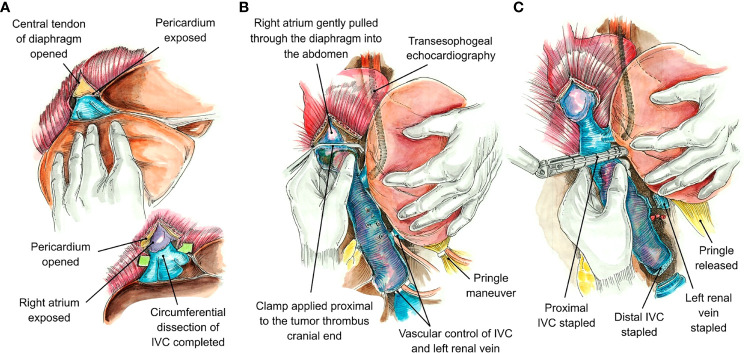
Tumor thrombus (TT) removal avoiding the use of cardiopulmonary bypass (CPB). The central tendon of the diaphragm is opened vertically, and the pericardium is exposed. If more exposure is required, the pericardium may also be opened. Circumferential control of the inferior vena cava (IVC) at a suprahepatic level facilitates TT removal **(A)**. The right atrium may be pulled downward through the diaphragm and into the abdomen, and the cranial end of the TT was controlled under Pringle maneuver conditions by cross-clamping the IVC above this level. Simultaneous transesophogeal echocardiography provides visual guidance during the performance of these maneuvers **(B)**. When the tumor thrombus can be milked down distal to the major hepatic vein orifices, an endo-GIA stapler may be utilized to staple the IVC proximally. Once the IVC is stapled, the liver vascular flow can be reestablished by releasing the Pringle maneuver. The procedure is completed by stapling the IVC distally and the left renal vein close to its entrance in the vena cava **(C)**.

If the TT extended to or above the diaphragm, the central tendon of the diaphragm was dissected to the supradiaphragmatic area until the intrapericardial IVC was fully exposed (**Figure 2**). The dissection was circumferential so that the intrapericardial IVC could be completely encircled below or above the confluence into the right atrium (RA). The RA was gently pulled beneath the diaphragm and into the abdomen. If more exposure of the RA was required, the central tendon of the diaphragm could be incised at the midline, allowing the pericardium to be exposed, and a pericardiotomy could be performed. Use of intraoperative transesophageal echocardiography (TEE) was critical to delineate the cranial extent and mobility of the TT during dissection of the retrohepatic IVC, supradiaphragmatic IVC, and RA and to confirm that there were no pulmonary artery emboli or TT extending into the RA. In addition, the intraoperative TEE acted as a guide during application of the vascular clamp onto the RA (if needed for proximal control due to the extent of TT), making sure that the clamp excluded the tumor and that the coronary sinus was not obstructed.

**Figure 3:**
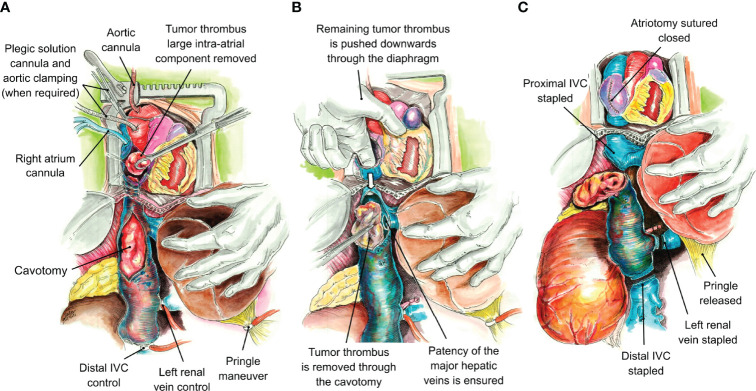
Tumor thrombus removal by means of cardiopulmonary bypass. Cannulae are placed in the aortic root and the right atrium. If cardiac arrest is required, a vascular clamp is placed at the aortic arch proximally, and an additional cannula is placed for plegic solution release. Pringle maneuver is initiated, a right atriotomy is performed, and the large component of the tumor thrombus occupying the right atrium is removed through the atriotomy **(A)**. The remaining tumor thrombus in the right atrium and proximal inferior vena cava is pushed downward through the atriotomy in a caudal direction. A longitudinal cavotomy permits the removal of the remaining tumor thrombus under direct visual control, ensuring the patency of the major hepatic veins *ostia* at the same time **(B)**. Once the cranial thrombus component is removed, the inferior vena cava is stapled below the entrance of the major hepatic veins, the atriotomy is sutured closed, and the Pringle maneuver is released to reestablish the vascular flow into the liver. The procedure is completed by stapling the inferior vena cava distally and the left renal vein close to its ostium **(C)**.

In cases of level IV thrombi with a large intra-atrial component not fully accessible from the abdominal field, the use of CPB was considered necessary. For this purpose, a midline sternum incision was used. Cannulation was performed in a standard fashion through the RA, right femoral vein, and distal ascending aorta (**Figure 3**). Proximal aortic clamping and additional cannulation were used for plegic solution administration when required. The type and parameters of CPB utilized were discretional upon the criteria of the cardiothoracic team involved and do not represent a constant along the study period, since a historical series is being reported here. Overall, CPB shifted from deep hypothermic under cardiac arrest to normothermic beating heart during the study period.

**Figure 4:**
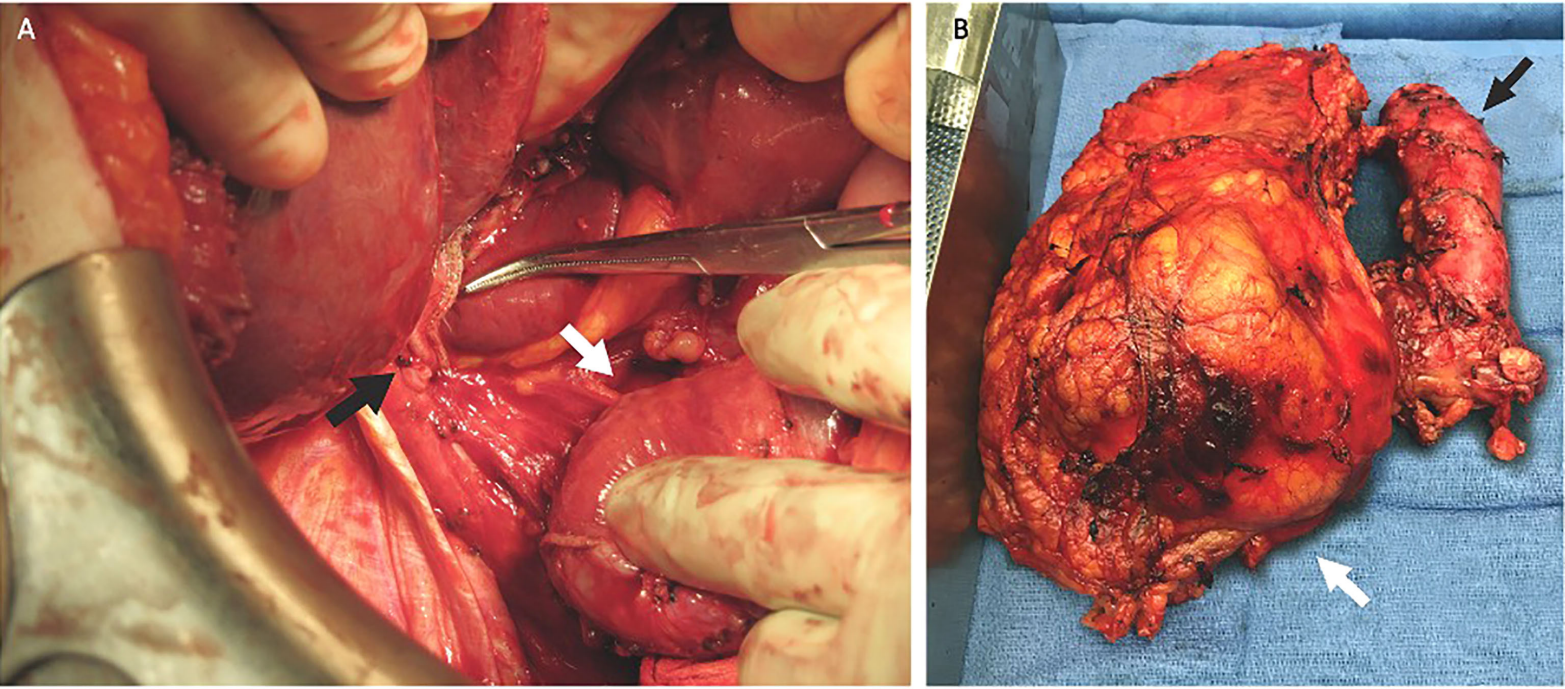
The inferior vena cava (IVC) was stapled at the level of the major hepatic veins (black arrow). The IVC with tumor thrombus (TT) is under the surgeon’s hand (white arrow) **(A)**. Surgical specimen of a large right renal tumor (white arrow) with the IVC completely stapled containing the TT (black arrow) **(B)**.

Once the liver and IVC were completely mobilized *via* the piggyback technique, vascular clamps were placed in the infrarenal IVC, followed by the left renal vein. The TT was then milked below the major hepatic veins, and the IVC was clamped without the need for a Pringle maneuver. If the TT was bulky, not freely mobile, and could not be milked downward out of the intrapericardial IVC, a Pringle maneuver was performed to temporarily occlude blood inflow to the liver. For level IV thrombi requiring a bypass, the thrombus was removed through an RA incision performed after CBP was initiated. Once the bulky atrial component was removed, the remaining proximal thrombus was pushed downward and removed through a cavotomy, ensuring complete thrombus removal and full patency of the major hepatic veins *ostia*. The cavotomy was sutured closed in its proximal segment, and the IVC was stapled just below the major hepatic veins and distal to the TT caudal end (**Figure 4**). The left renal vein was either stapled or oversewn. At the end of the resection, TEE was repeated to rule out any pulmonary artery emboli or piece of TT left behind.

## Results

Twenty-two patients were included in the study. Patients’ demographics, tumor characteristics, and perioperative variables are described in **Table 1**. Median age at surgery was 62.5 (range: 45–79) years, and 19 (86%) of the patients were men. Majority of the patients (N = 14, 64%) had a level III thrombus (IIIa, n = 3; IIIb, n = 6; IIIc, n = 3; IIId, n = 2). Seven patients (32%) had a level IV thrombus, and one patient (5%) had a level II thrombus. Median tumor size was 12 (range: 4–22) cm.

**Table 1:** Patient demographics, tumor characteristics, and perioperative factors.

All	22 (100%)
**Sex**	
Men	19 (86%)
Women	3 (14%)
**Age (years)**	
Median (range)	62.5 (45–79)
**Thrombus level**	
II	1 (5%)
III	14 (64%)
IIIa	3 (14%)
IIIb	6 (27%)
IIIc	3 (14%)
IIId	2 (9%)
IV	7 (32%)
**Tumor size (cm)**	
Median (range)	12 (4–22)
**Estimated blood loss (ml)**	
Median (range)	1,350 (200–25,000)
**Units of packed red blood cells transfused**	
Median (range)	3.5 (0–30)
**Cardiopulmonary bypass (CPB) performed**	
No	18 (82%)
Yes	4 (18%)
**Pathology**	
Clear cell	18 (82%)
Papillary	2 (9%)
Mixed	2 (9%)
**Stage**	
**Pathologic T stage**	
T3b	12 (55%)
T3c	10 (45%)
**Pathologic N stage**	
N0	8 (36%)
N1	7 (32%)
N2	2 (9%)
Nx	5 (23%)
**Clinical M stage**	
M0	4 (18%)
M1	6 (27%)
Mx	12 (55%)
**Complications (Clavien–Dindo grade)**	
0	9 (41%)
I	2 (9%)
II	8 (36%)
IIIa	1 (5%)
IIIb	0 (0%)
IVa	1 (5%)
IVb	1 (5%)
**Length of stay (days)**	
Median (range)	11 (5–50)
**Death during follow-up**	
No	18 (82%)
Yes	4 (18%)
**Time-to-death (years) during follow-up (N = 4)**	
Median (range)	0.9 (0.1–1.3)
**Follow-up time (years) among ongoing survivors (N = 18)**	
Median (range)	1.5 (0.5–7.0)

Intraoperatively, median estimated blood loss was approximately 1.35 (range: 0.2–25) L, and patients were transfused a median of 3.5 (range: 0–30) units of packed red blood cells. Surgery was completed without CPB in 18 (82%) of the cases. For patients requiring CPB, two (9%) had an intraoperative cardiac arrest, while two (9%) did not have cardiac arrest. All patients requiring CPB had a level IV TT.

Majority of the patients had clear cell RCC (N = 18, 82%). Two patients (9%) had papillary RCC, and two patients (9%) had unclassified cell pathology. Five patients (28%) had clear cell with differentiation. Of those, two patients had rhabdoid differentiation, two patients had sarcomatoid differentiation, and one patient had rhabdoid and sarcomatoid differentiation. Twelve patients (55%) were classified as TNM stage T3b, and 10 patients (45%) were TNM stage T3c. Eight patients (36%) had no nodal metastasis on pathology, while 9 patients (41%) had Stage 1 or 2 nodal metastasis, and 5 patients (23%) had an unknown status for nodal metastasis.

Clavien–Dindo grades of postoperative complications are presented in **Table 1**. Complications were observed in 13 (59%) cases, 10 (77%) of which were Clavien–Dindo grades I–II. Of the remaining three cases, one patient had a grade IIIa complication, requiring chest tube placement under local anesthesia for a pleural effusion. Another patient with a level IV TT suffered a grade IVa complication including postoperative bleeding from the duodenum requiring reintervention for hemostasis, *Clostridioides difficile (C. difficile)* infection, sepsis, and prolonged intensive care unit stay. Finally, in another case of a level IV TT that required CPB, the patient suffered a grade IVb complication with cardiogenic shock, pneumonia, and subsequent death at 44 days after surgery, which was preceded by postoperative evisceration requiring abdominal wall repair with mesh. Of note, none of the patients presented with a postoperative complication as a result of pulmonary emboli or dislodgment of the TT.

Median length of stay was 11 (range: 5–50) days. Four patients died (range: 0.1–1.3 years), with one being due to a postoperative complication (described above) and the other 3 deaths being due to disease progression. Median postoperative follow-up among 18 ongoing survivors (at last follow-up) was 1.5 (range: 0.5–7.0) years.

Perioperative creatinine trends are shown in **Table 2**. Median preoperative creatinine was 1.2 (range: 0.4–2.7) mg/dl. This increased slightly after surgery, with a median postoperative creatinine of 1.3 (range: 0.9–2.2) mg/dl. Twenty-one patients had a creatinine result available at 6 months postoperatively, and 15 patients had a creatinine result available at 12 months postoperatively. Median creatinine at 6 months of follow-up was 1.30 (range: 0.5–1.8) mg/dl and at 12 months of follow-up was 1.40 (range: 0.6–2.0) mg/dl. Student’s t-tests of the mean change in ranked serum creatinine between baseline values and immediate postoperative and 6- and 12-month postoperative values found no significant changes in renal function over time (P > 0.20 for each test). No patient required renal replacement therapy as of last follow-up.

**Table 2:** Preoperative, perioperative, and postoperative creatinine levels.

		N (missing)
**Preoperative creatinine (mg/dl)**		
Median (range)	1.2 (0.4–2.7)	20 (2)
**Discharge creatinine (mg/dl)**		
Median (range)	1.3 (0.9–2.2)	22 (0)
**Creatinine at 6 months postoperative (mg/dl)**		
Median (range)	1.3 (0.5–1.8)	21 (1)
**Creatinine at 12 months postoperative (mg/dl)**		
Median (range)	1.4 (0.6–2.0)	15 (7)

## Discussion

RCC with TT extending into the intrahepatic IVC or the RA are technically challenging surgical cases, although complete resection remains a mainstay of treatment to improve overall survival (1, 2, 5, 6, 16). When performing right radical nephrectomy and IVC thrombectomy with a level II–IV obstructing TT, this review demonstrates that ligation of the left renal vein may safely be performed without adversely impacting renal function. Preserved renal function is demonstrated both in the immediate postoperative period and during follow-up, allowing patients to avoid renal replacement therapy and potential complications associated with IVC reconstruction.

While the right renal vein rarely develops adequate collaterals to redistribute venous return, the left renal vein develops sufficient collateral drainage in the setting of long-standing obstruction of the IVC through the left gonadal and adrenal veins, azygos–hemiazygos system, and occasionally through lumbar veins (which may be present on the posterior aspect of the left renal vein) (7–9, 16, 17). Of note, ligation of the right renal vein is generally not safe because sufficient collateral drainage does not develop. Division of the left renal vein in the absence of adequate collateral drainage can result in impairment of renal function postoperatively (such as in abdominal aortic aneurysm repair) (18, 19). Adequate collateral drainage and extensive bland thrombus can be identified on preoperative imaging, and patients are not expected to have lower extremity edema on examination (9, 20, 21). When it is unclear if adequate collateral drainage is present, it has been suggested that a clamp trial may be performed intraoperatively before division of the left renal vein. Tense engorgement within 3 min of clamping may indicate insufficient collateral drainage (18).

This surgical approach represents a safe option that may also be used when using a robot-assisted approach (22, 23). It simplifies the procedure, since diversion of the left renal vein into another vessel (such as the inferior mesenteric vein) or IVC reconstruction through grafting is not required (17), thus avoiding operative time-consuming procedures and the need for prolonged antibiotic and anticoagulation therapy to prevent graft infection and/or occlusion (particularly frequent in the setting of hypercoagulability of malignancy) (17, 24). Although robotic assistance allows for increased precision in instrument management, smaller incisions, decreased blood loss, less pain, and earlier recovery (25, 26), the need to rely on other specialists for damage control in the event of a possible complication is still necessary. The distance of the surgeon from the operating table may be a disadvantage at the time of a theoretical conversion to open surgery, which can be a problem during the management of higher-level TT, whose inadvertent fragmentation often has devastating consequences for the patient. The safe robotic management of higher-level TT remains an area of investigation.

Although this is the largest experience available to date in the literature for circumferential IVC resection without reconstruction for right-sided RCC with level II–IV TT, these cases remain rare even in referral centers, limiting the sample size. Preoperative creatinine was not available for two patients who underwent surgery before implementation of an electronic medical record. Since many of these patients are referred to a tertiary center for surgery, postoperative creatinine was not always available, since some patients are followed postoperatively at their home institution. This series includes patients from two centers where the surgical approach has been standardized. Nevertheless, the outcomes reported must be handled cautiously, since they may not be reproducible by surgical teams of different volumes and experiences.

For patients with right-sided RCC with obstructing level II–IV IVC thrombus, ligation of the left renal vein and resection of the IVC without reconstruction do not appear to adversely impact mid-term renal function.

## Data Availability Statement

The raw data supporting the conclusions of this article will be made available by the authors without undue reservation. 

## Ethics Statement

This study was reviewed and approved by the University of Miami IRB (20200791) and abides by the Helsinki Declaration (as revised in 2013). The University of Miami IRB’s decision to waive consent to participate required that the IRB finds and documents that: 1) The research involves no more than minimal risk to the subjects; 2) The waiver or alteration will not adversely affect the rights and welfare of the subjects; 3) The research could not practicably be carried out without the wavier or alteration; and 4) Whenever appropriate, the subjects will be provided with additional pertinent information after participation.

## Author Contributions

Conception and design: GC. Acquisition of data: GC, LH, MR-C, FH-A, CH and MMT. Analysis and interpretation of data: JG, LH, JJG, and GC. Writing of the article: LH, MMT, JG and RS. Statistical analysis: JJG. Supervision: GC and JJG. All authors contributed to the article and approved the submitted version.

## Conflict of Interest

The authors declare that the research was conducted in the absence of any commercial or financial relationships that could be construed as a potential conflict of interest.

## Publisher’s Note

All claims expressed in this article are solely those of the authors and do not necessarily represent those of their affiliated organizations, or those of the publisher, the editors and the reviewers. Any product that may be evaluated in this article, or claim that may be made by its manufacturer, is not guaranteed or endorsed by the publisher.
